# ADHD and the Inflammatory Pathway: from infections to inflammations

**DOI:** 10.1192/j.eurpsy.2025.172

**Published:** 2025-08-26

**Authors:** I. Manor

**Affiliations:** 1 ADHD clinic, Geha MHC, Petach-Tiqva; 2 psychiatric division, Faculty of Medicine and Health-ralted disorders, Tel Aviv University, Tel Aviv, Israel

## Abstract

**Abstract:**

Attention-Deficit/Hyperactivity Disorder (ADHD) has been reported as a risk factor for COVID-19 infection and severity of illness. This presentation outlines the research trajectory that began during COVID-19 studies and has since demonstrated that ADHD is not only associated with an increased risk of infection but also with long-term COVID-19 syndrome (LCS). The chain of studies supporting these findings includes the association of ADHD with infections and inflammatory/autoimmune disorders, such as Glucose-6-Phosphate Dehydrogenase (G6PD) deficiency and Familial Mediterranean Fever (FMF). These links, alongside globally reported associations between ADHD and other autoimmune disorders, such as Systemic Lupus Erythematosus (SLE), underscore the potential involvement of inflammatory and immune dysregulation in the pathophysiology of ADHD. The emerging data linking ADHD with inflammatory conditions—at both clinical and genetic levels—represents a significant new direction in ADHD research. Further investigation into these connections may provide deeper insights into the underlying mechanisms of ADHD and contribute to developing novel therapeutic strategies.

**Disclosure of Interest:**

I. Manor Consultant of: Last three years: Teva Israel Ltd., Madison Israel Ltd., Takeda Israel Ltd., Peri Ltd., Mindtension Ltd.,

